# Efficacy of Leuprorelide acetate (Eligard®) in daily practice in Brazil: a retrospective study with depot formulations in patients with prostate cancer

**DOI:** 10.1590/S1677-5538.IBJU.2019.0212

**Published:** 2020-02-20

**Authors:** Carla S. M. de Freitas, Aleida N. Soares

**Affiliations:** 1 Hospital do Câncer de Muriaé MG Brasil Hospital do Câncer de Muriaé, MG, Brasil; 2 Instituto de Ensino e Pesquisa da Santa Casa de Belo Horizonte Belo HorizonteMG Brasil Instituto de Ensino e Pesquisa da Santa Casa de Belo Horizonte, Belo Horizonte, MG, Brasil

**Keywords:** Prostatic Neoplasms, agonists [Subheading], Prostate-Specific Antigen

## Abstract

**Introduction:**

Androgen deprivation therapy (ADT) is the mainstay of therapy for advanced prostate cancer. Studies addressing the efficacy of different depot formulations of long acting luteinizing hormone releasing hormone agonists in the Brazilian population are lacking. We aimed to compare the efficacy of three schedules of leuprolide acetate in lowering PSA in a real world population.

**Materials and Methods:**

We reviewed the medical records of patients with prostate cancer seen at our institution between January 2007 and July 2018. We analyzed patients treated with long-acting leuprolide acetate and grouped these patients into three strata according to the administration of ADT every 1, 3 or 6 months. The primary outcome was the serum prostate specific antigen (PSA) levels at 6 and 12 months after treatment initiation. We used Friedman test to compare the distribution of PSA levels at baseline and at 6 and 12 months within each treatment stratum. We considered two-sided P values <0.05 as statistically significant. We analyzed toxicity descriptively.

**Results:**

We analyzed a total of 932 patients, with a median age of 72 years and a median time since diagnosis of prostate cancer of 8.5 months. ADT was administered monthly in 115 patients, quarterly in 637, and semiannually in 180. Nearly half of the patients had locally advanced disease. In comparison with baseline, median serum PSA levels were reduced at 12 months by at least 99.7% in the three strata (P <0.001 in all cases). Sexual impotence and hot flashes were the most frequently reported toxicities.

**Conclusion:**

To our knowledge, this is the largest assessment of real-world data on alternative schedules of leuprolide in a Brazilian population. Our study suggests that PSA levels can be effectively be reduced in most patients treated with monthly, quarterly, or semiannual injections of long-acting leuprolide acetate.

## INTRODUCTION

According to GLOBOCAN, it is estimated that 1.276.106 men were diagnosed with prostate cancer worldwide in 2018, with an expect 6.3% increase in incidence for the year of 2020 (1-3). By the year 2030, the burden from prostate cancer in Central and South America is expected to nearly double as a result of population growth and aging, moreover, increased early detection and public awareness are likely to lead to further increase in incidence in this world region (4). In Brazil, prostate cancer is the most frequent non--cutaneous tumor and the second leading cause of death from cancer in men (5).

Given the androgen dependence that characterizes prostate cancer, androgen deprivation therapy plays a key role in distinct phases of the disease. Among patients with advanced disease, androgen deprivation is the mainstay of therapy for both hormone-sensitive and castration-resistant prostate cancer (6), with long-acting luteinizing-hormone-releasing hormone (LHRH) agonists being currently the main form of achieving androgen deprivation (7). Most patients treated with a LHRH agonist achieve castrate testosterone levels similar to those found after bilateral orchiectomy (8). As a result of testosterone suppression, disease control can be achieved in the majority of patients as indicated, for example, by decreased levels of prostate specific antigen (PSA). Clinical trials of different LHRH agonists have demonstrated the efficacy of these agents in different settings. In particular, the efficacy and safety of Eligard^®^ (a depot formulation of leuprolide acetate for subcutaneous injection every 1, 3 or 6 months) have been assessed and confirmed in clinical trials (9, 10). However, such studies are often limited by strict selection criteria, and a need remains for “real-world” data collected in observational studies (9-14).

## OBJECTIVES

In the current study, we sought to investigate the efficacy of Eligard^®^ used monthly, quarterly and semiannually, in a heterogeneous population of patients from routine clinical practice at the Muriaé Cancer Hospital, state of Minas Gerais, Brazil.

## MATERIALS AND METHODS

### Study design and patient eligibility

The study protocol was reviewed and approved by the Institutional Ethics Committee, and written informed consent was waived due to the observational nature of the investigation. In this retrospective study, we reviewed the medical records of 932 patients with prostate cancer seen at our institution between January 2007 and July 2018. Given the observational nature of the study, diagnostic and treatment decisions, including the choice of androgen deprivation therapy, formulation and schedule, as well as use of concomitant medication, were at the discretion of the attending physicians.

Eligible patients were men with prostate cancer, aged ≥18 years old, and treated with Eligard® (henceforward referred to as androgen de-privation) at some point during the study period. We included in this study all the patients who had baseline serum PSA results. We grouped patients into three separate strata according to the administration of androgen deprivation therapy every 1, 3 or 6 months.

### Data collection, outcomes of interest, and statistical analysis

We collected data on demographic patient characteristics, features of prostate cancer, dates and clinical events related to treatment, serum PSA results, and toxicity. The primary outcome measure was the serum PSA levels at 6 months and 12 months after treatment initiation. After using the Kolmogorov-Smirnov test to demonstrate that PSA levels did not have normal distribution, we used Friedman test to compare, within each treatment stratum (monthly, quarterly, or semiannually), the distribution of PSA levels at baseline and at 6 and 12 months. Moreover, we used Wilcoxon's rank sum test to make pairwise comparisons between adjacent time points within strata. We made no comparisons between strata. We considered two-sided P values <0.05 as statistically significant and performed the analyses with SPSS, version 22.0.

## RESULTS

### Patient characteristics

A total of 932 patients fulfilled the selection criteria and were analyzed, with their key characteristics being displayed in Table-1. The median age was 72 years (interquartile range, 65 to 78 years). Comorbidities were reported in 43.8% of the study population, most of which cardiovascular (85.0%). The median time since diagnosis of prostate cancer was 8.5 months, with 598 (64.2%) patients being diagnosed ≤12 months and 158 (17.0%) >4 years. The Gleason score was distributed relatively evenly between, low (Gleason ≤6), intermediate (Gleason 7) and high (Gleason ≥8) grade, and nearly three quarters of patients had non-metastatic disease. Bone metastases were reported in 161 (74%) of the patients in stage IV, while 5 patients (2.3%) had metastases at other distant sites and 12 (5.5%) had lymph node metastases. Radiotherapy and radical prostatectomy were the most frequent treatment modalities, in the beginning of the use of Eligard®.

**Table 1:** Patient characteristics (n=932).

Characteristic			Value
**Age, years**			
	Median (interquartile range)		72 (65 to 78)
**Time since diagnosis of prostate cancer in months**			
	Median (interquartile range)		8.5 (5 to 26)
**Ethnicity**			
	White		307 (32.9%)
	Black		619 (66.5%)
	Not available		6 (0.6%)
**Gleason score**			
	Low		320 (34.3%)
	Intermediate		318 (34.1%)
	High		274 (29.4%)
	Not available		20 (2.1%)
**Prostate cancer stage at diagnosis**			
	I		35 (3.8%)
	II		369 (39.6%)
	III		269 (28.9%)
	IV		218 (23.4%)
	Not available		41 (4.4%)
**Treatment**			
	Radical prostatectomy		163 (17.5%)
	Transurethral resection of the		72 (7.7%)
	prostate		
	**Radiotherapy**		588 (63.1%)
		RT locally advanced	235 (39.9%)
		RT rescue	104 (17.7%)
		RT adjuvant	122 (20.7%)
		RT palliative	127 (21.7%)
Chemotherapy			52 (5.6%)
Not available			57 (6.1%)

### Indication for androgen deprivation

Androgen deprivation therapy was administered monthly in 115 patients, quarterly in 637, and semiannually in 180. Overall across strata, 711 (76.3%) of the patients were treated concomitantly with other modalities, and 129 (13.8%) patients had received previous hormone therapy for the underlying disease. Nearly half of the patients were considered to have locally advanced prostate cancer, and this was the main indication for androgen deprivation. There was biochemical recurrence after prostatectomy in 104 of the 235 (44.3%) patients who underwent prostatectomy and in 122 (20.7%) of the 588 treated with radiotherapy. RT locally advanced (235, 39.9%), rescue radiotherapy 104 (17.7%), adjuvant radiation therapy 122 (20.7%), palliative radiotherapy 127 (21.7%).

Among the 932 patients, 803 (86.2%) were treated with the LHRH agonist alone, while 129 (13.8%) of the patients received it in combination with an antiandrogen (bicalutamide [8.4%], flutamide [4.8%], or cyproterone acetate [0.6%]).

### PSA levels

**Figure 1:**
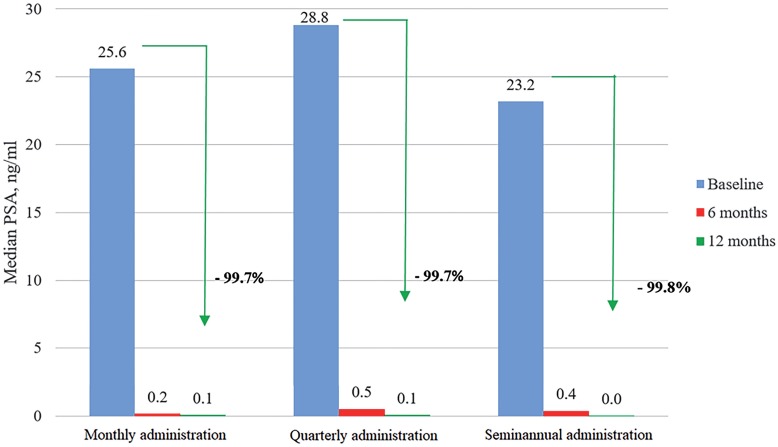
Median levels of serum prostate specific antigen (PSA) in each stratum over time.

Table-2 presents summary results for the serum PSA levels in each stratum at the three time points of interest. In comparison with baseline, median serum PSA levels were reduced at 12 months by at least 99.7% in the three strata (arrows in Figure-1). As shown in Figure-1, all pairwise comparison between adjacent time points within strata were statistically significant, with the exception of the comparison between 6 and 12 months for the monthly administration.

**Table 2:** Serum prostate specific antigen (PSA) levels versus leuprolide schedule.

Stratum	Median PSA (interquartile range), ng/mL			P value
	Baseline	6 months	12 months	
Monthly	n=115	n=108	n=104	<0.001
	25.6 (13.7 to 86.0)	0.18 (0.05 to 1.6)	0.08 (0.1 to 0.7)	
Quarterly	n=637	n=588	n=512	<0.001
	28.8 (13.0 to 87.0)	0.54 (0.05 to 3.59)	0.09 (0.01 to 1.0)	
Semiannualy	n=180	n=151	n=103	<0.001
	23.2 (9.4 to 70.5)	0.39 (0.04 to 2.08)	0.04 (0.01 to 0.37)	

### Safety and tolerability

A total of 72 (7.7%) patients reportedly had treatment-associated toxicity registered in the medical records. Among these cases, sexual impotence and hot flashes were the most frequent and were reported in 58 (6.2%) and 23 (2.5%) patients, respectively. More severe toxicity that was possibly associated with treatment was reported in only 9 (0.9%) of patients: there were two cases of hemorrhagic stroke, and one case each of heart failure secondary to coronary insufficiency, peripheral vascular insufficiency, angina, pulmonary embolism, and acute myocardial infarction. Despite this potential association with treatment, all these cases had comorbidity whose causal link with the toxicity could not be ruled out by chart review. In many cases, treatment for the comorbidity was reportedly used irregularly by the affected patients. No patient reportedly discontinued treatment prematurely due to toxicity.

## DISCUSSION

To our knowledge, the current study is the largest reported assessment of real-world data on patients with prostate cancer in Brazil. The study confirms the effectiveness and safety of this depot formulation of leuprolide acetate in clinical practice. Effectiveness was assessed on the basis of serum PSA declines during the first year of treatment, and safety was ascertained on the basis of toxicity reported in the medical records. Although the reliability and state of completion of medical records are well-known limitations of retrospective studies, the toxicity profile disclosed by our study overlaps with the profile of adverse events reported in clinical trials. On the other hand, the PSA data are objective and were available for the vast majority of time points. As a result, we believe our results add to the current literature by confirming the effectiveness of this LHRH agonist and the validity of a flexible approach, in terms of the choice of administration schedule, which may suit individual patients and clinicians according to their priorities.

Three other observational studies have been conducted with the objective of evaluating the effectiveness, tolerability and/or impact on the quality of life of this depot formulation of leuprolide acetate in daily clinical practice among patients with prostate cancer. In the ELIRE study, conducted in France, the formulation used was for administration every 3 or every 6 months (11). Among 1.853 registered patients, the mean age was 75 years, and the mean time to diagnosis was 7 months. Interestingly, the criteria for choosing between the quarterly or semiannual administration were different, with patients using the latter schedule being more likely to be older and less autonomous than those using the former. The authors concluded that semiannual administration provides more flexibility in the management and follow-up of patients with locally advanced or metastatic prostate cancer. The results suggest that the semiannual use of the LHRH agonist is as effective as the other regimens, resulting in a difference in treatment cost between groups, impacting positively on Brazilian public health. The MANTA study, conducted in Belgium, assessed both a monthly and a quarterly schedule in 243 patients and confirmed that both depot formulations of leuprolide acetate are well tolerated and reliably lowered serum PSA and testosterone levels in routine clinical practice (13). Finally, a study in Germany assessed the administration of leuprolide acetate every 6 months in 1.273 patients (12). At 12 months, there was a median reduction of 96% (to 0.5ng/mL) in serum PSA levels. Interestingly, further PSA and serum testosterone decreases were observed in a subpopulation of patients treated initially with other LHRH analogues and who switched to 6-monthly leuprolide acetate. Similar to the present study, toxicity was reported in the German study in 9% of patients, and the majority were not considered serious. Of note, this LHRH agonist was found to be the most frequently used form of medical castration in another study from Germany, in this case based on a claims database (15).

Clinical trials usually demonstrate the efficacy and tolerability of therapeutic agents in relatively homogeneous populations of patients meeting strict selection criteria. Real-world studies are important because they have broader criteria and may include far more patients in populations that are likely to be observed in routine clinical practice. Compared with patients enrolled in clinical trials (9, 10), patients in observational studies usually display larger variability in tumor staging, Gleason scores, indications for androgen deprivation, and comorbidity. Many of the patients analyzed in observational studies, including ours, would have been excluded from clinical trials (11-13). Reassuringly, however, the results of clinical trials and observational studies with this LHRH analogue overlap to show that PSA levels can be effectively reduced in most patients treated with monthly, quarterly, and semiannual injections.

Our study did not aim at formally to compare the effectiveness or tolerability of the three schedules of administration of this LHRH analogue. Nevertheless, the results show that median serum PSA levels were reduced by at least 99.7% across the three strata, with no notable differences among them. Moreover, these results were seen in spite of the fact that most patients received the LHRH analogue alone, unlike in some studies, in which variable proportions of patients were treated with combinations containing an anti-androgen, a bisphosphonate, or chemotherapy. In such a heterogeneous population as ours, and considering the ease of use and local tolerability of this depot formulation, we believe that the choice among the different schedules has to be individualized based on preference and health-care system convenience. Arguably, semiannual injections can provide benefits in terms of patient visits and use of resources, but this hypothesis remains to be tested formally in our specific health-care scenario. In Japan, for example, the 6-month formulation was found to reduce medical costs, loss of productivity, and intangible costs in comparison with the 3-month formulation (16). Likewise, in a cost-minimization analysis conducted in Europe, the 6-month formulation was found to offer the greatest cost savings, and the authors considered that it should be the treatment of choice in eligible European patients (17). In the Brazilian health-care system, which aims at providing universal coverage for all citizens (18), it is conceivable that a reduced number of patient visits, especially for the elderly and for those living far from the treatment center, may bring efficiencies and allow for the opening of vacancies for other patients in need.

## CONCLUSIONS

Our study confirms that serum PSA levels can be effectively reduced in most of the patients with prostate cancer treated with monthly, quarterly, or semiannual injections of this LHRH agonist. This therapeutic goal is achieved at the expense of a relatively favorable toxicity profile, and it is hoped that schedules of administration every 6 months will bring the added benefit of convenience and cost savings in clinical practice in Brazil and elsewhere.
